# The Relationship Between Loneliness and Sleep Disturbances: The Roles of Community Resilience, Social Support, Anxiety, and Substance Use Coping

**DOI:** 10.3390/ijerph23081014

**Published:** 2026-08-03

**Authors:** Hwanseok Winston Choi, Michelle Brazeal, Joohee Lee

**Affiliations:** 1 Department of Community Health & Preventive Medicine, Morehouse School of Medicine, Atlanta, GA 30310, USA; 2 School of Social Work, University of Southern Mississippi, Hattiesburg, MS 39406, USAjoohee.lee@usm.edu (J.L.)

**Keywords:** anxiety, community resilience, loneliness, sleep disturbances, social support, South Mississippi

## Abstract

**Highlights:**

**Public health relevance—How does this work relate to a public health issue?**

- Chronic sleep insufficiency affects over one-third of the adult population and is directly linked to severe public health outcomes, including cardiovascular disease, obesity, impaired workplace safety, and an estimated billions of dollars in annual healthcare costs and lost productivity.
- By identifying community resilience as a protective factor against loneliness and subsequent anxiety, this study moves sleep health interventions beyond clinical, individual-focused treatments (like sleep hygiene or sleep medication) toward community-level public health strategies and social infrastructure investments.

**Public health significance—Why is this work of significance to public health?**

- This research highlights community resilience and perceived social support as critical, modifiable upstream factors that are inversely associated with loneliness and anxiety, providing public health officials with concrete social structural targets to improve population-level sleep and mental health.
- By demonstrating that anxiety fully mediates the relationship between loneliness and sleep disturbances, this study provides a framework for multi-layered public health initiatives, such as combining community disaster preparedness with targeted mental health screenings, especially in ecologically vulnerable regions like coastal communities.

**Public health implications—What are the key implications or messages for practitioners, policy makers, and/or researchers in public health?**

- Public health policies must treat community development and emergency preparedness infrastructure as direct investments in mental and physical health. Enhancing community resilience, such as strengthening local information channels and neighborhood networks, serves as a scalable, non-clinical intervention that reduces population-wide loneliness and protects sleep health.
- Clinical and community public health practitioners should shift from isolated sleep-hygiene campaigns to integrated, multi-level interventions. Because anxiety fully drives the relationship between social isolation and sleep disturbances, screening and treating underlying psychological distress must be paired with initiatives that rebuild an individual’s local social support systems.

**Abstract:**

This study examined the relationship between loneliness and sleep disturbances and explored the roles of community resilience and psychosocial factors (social support, anxiety, and substance use coping) in shaping this relationship. A spatially stratified random sampling approach was applied to select households between Interstate 10 and the Gulf of Mexico coastline across three Mississippi counties: Hancock, Harrison, and Jackson. Amos version 30 was used to analyze the proposed structural equation model (SEM) based on responses from 310 participants (mean age = 50.47 years, SD = 17.57; 53.3% female). Data were collected using validated self-report instruments, including the Communities Advancing Resilience Toolkit (CART), the Multidimensional Scale of Perceived Social Support (MSPSS), the UCLA 3-item Loneliness Scale, the GAD-2, and the Brief COPE. Results revealed that initially, a significant association was observed between loneliness and sleep disturbances. However, this direct association was non-significant upon the inclusion of anxiety and substance use coping, showing instead a statistically significant indirect association operating through anxiety symptoms. Higher levels of loneliness were associated with increased anxiety symptoms, which in turn were associated with higher sleep disturbances. No indirect relationship between loneliness and sleep disturbances through substance use coping was found. The results also found that higher levels of community resilience and social support were associated with lower levels of loneliness. These findings highlight the need for multilevel strategies to improve sleep quality, which creates a nested system in which community strengths and individual support systems are jointly positioned as upstream correlates of lower individual isolation.

## 1. Introduction

Sleep is a cornerstone of human health and well-being. Sleep not only represents a state of unconsciousness; it is a vital physiological process that plays a critical role in maintaining overall health and functioning. It is during sleep that the body engages in essential restorative processes, repairing tissues, consolidating memories, and regulating various bodily functions [1]. In addition, adequate sleep is crucial for cognitive functioning and emotional regulation [2,3].

However, this essential foundation is often disrupted, as a significant percentage of adults experience insufficient sleep, with statistics indicating that the prevalence remained steady between 33% and 37% from 2013 to 2022 [4].

Sleep quality appears to be influenced by multiple factors, including medical conditions, mental disorders, and social factors [5,6]. Loneliness has gained significant attention for its critical role in sleep quality [7,8,9], given that loneliness and social isolation are increasingly prevalent in contemporary society [10]. Nevertheless, research remains limited regarding the interplay of psychosocial and community factors in explaining the relationship between loneliness and sleep disturbances. This study examines the relationship between loneliness and sleep disturbances and explores the roles of community resilience and psychosocial factors (social support, anxiety, and substance use coping) in shaping this relationship, with implications for both theoretical knowledge and intervention.

### 1.1. Literature Review

#### 1.1.1. Loneliness, Anxiety, and Substance Use Coping as Predictors of Sleep Disturbances

There is no single definition that describes the experience of being lonely. At its simplest, it can be defined as “A state of solitude or being alone.” However, research has shown that individuals can be around others and still feel lonely. Feeling isolated, disconnected, a sense of inadequate social relationships, or an inability to find meaning can also represent loneliness [11].

Individuals who experience loneliness are more likely to report lower quality sleep [12] and more wakefulness after falling asleep [8,12]. These findings are further supported by a systematic review and meta-analysis study, which found that loneliness is associated with poorer sleep quality [7]. In addition, prior research suggests that this relationship may be mediated by other variables. Grey et al. found that the relationship between loneliness and sleep quality is both direct and indirect, mediated by anxiety [13].

Anxiety can be defined as either an emotion or a disorder. Feelings of anxiety can be defined as an uneasy feeling, often accompanied by restlessness, rapid breathing, gastrointestinal difficulties, or problems concentrating [14]. These symptoms can negatively impact sleep.

However, the relationship appears to be bidirectional [15,16]. Worry and accompanying physical symptoms can make it difficult to fall asleep and stay asleep, while the lack of quality sleep can add physical stress to the body that worsens the impact of stress. Studies have shown that sleep disturbances almost double the risk of experiencing anxiety symptoms, and anxiety increases sleep disturbances by 20% [16].

Coping can be defined as “the person’s constantly changing cognitive and behavioral efforts to manage specific external and/or internal demands that are appraised as taxing or exceeding the person’s resources” [17], p. 993. Sleep studies have also explored the role of coping strategies in sleep. Laudie et al. [18] found endorsement of substance use was associated with negative sleep quality during the pandemic. Similarly, Hernandez and Griggs [19] found that greater substance use was associated with shorter sleep duration, although this relationship appears to vary by substance type.

While the direct associations between individual loneliness and sleep disturbances are well-established in prior meta-analyses, existing models largely overlook how these pathways operate within regions exposed to chronic environmental stress. This study addresses that gap by using a spatially stratified sample from the disaster-prone Mississippi Gulf Coast to explore how these factors, such as community resilience and social support, interact with individual clinical symptoms. By positioning this inquiry within an ecologically vulnerable zone, the research moves beyond simple geographic replication to demonstrate how communal capacity can serve as a broad foundational buffer against individual psychological and physiological vulnerabilities.

Investigating these pathways using data established before the COVID-19 pandemic provides a necessary structural baseline. The pandemic introduced severe, acute, and globally uniform psychosocial disruptions that heavily confound contemporary epidemiological tracking. Examining a pre-pandemic cohort allows us to isolate the baseline mechanisms through which community-level infrastructure protects individual sleep and mental health under localized, chronic ecological stress without the compounding, extraordinary noise of a global health crisis.

#### 1.1.2. Community Resilience and Social Support as Predictors of Loneliness

When loneliness is understood as a psychological state of social isolation, feelings of being alone, and perceived disconnection, community and individual support systems may serve as protective or risk factors that influence individuals’ likelihood of experiencing loneliness.

Community resilience is not a single concept but a set of factors that describe how a community functions, particularly during stressful events. However, there are some common components used to define community resilience. These include “local knowledge, community networks and relationships, communication, health, governance and leadership, resources, economic investment, preparedness, and mental outlook” [20], para 3. Highly resilient communities are more likely to provide the systems and structures needed to support their members [21], leading to increased perceived social support and reduced loneliness [22].

Social support can be defined as a resource that others provide to an individual, including emotional, instrumental, informational, or companionship support [23] that can be categorized as formal or informal. Informal support includes family, friends, and significant others, while formal support includes structured and specialized support [24,25]. However, the size of the network or the provision of support does not necessarily correspond to an individual’s satisfaction with the support received. Perceived social support can be a more proximal indicator of psychological outcomes [26]. A substantial body of research indicates that greater perceived social support is associated with lower levels of loneliness [26,27,28].

### 1.2. The Current Study

**Figure 1:**
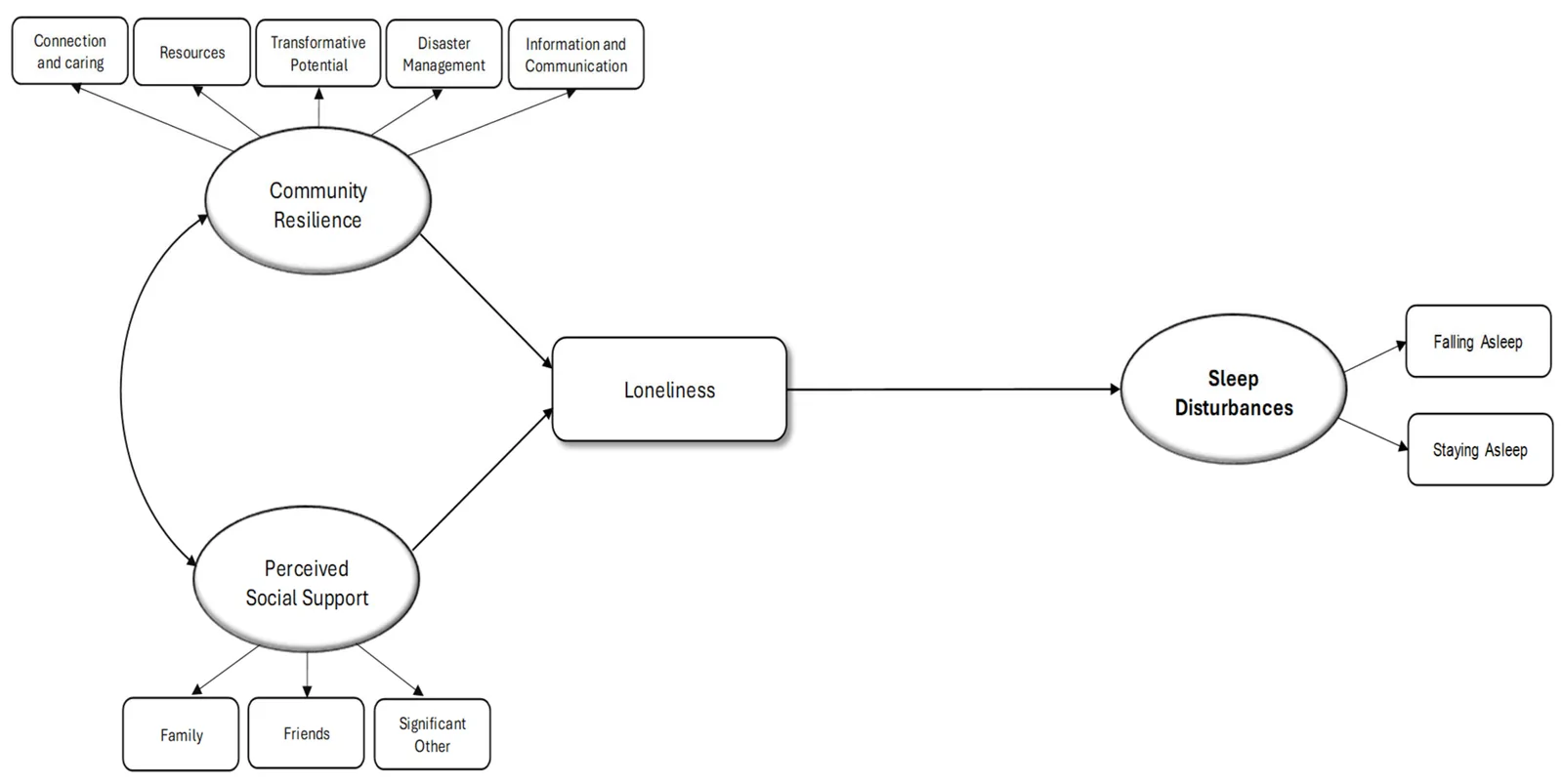
Proposed structural equation model of loneliness and sleep disturbances.

**Figure 2:**
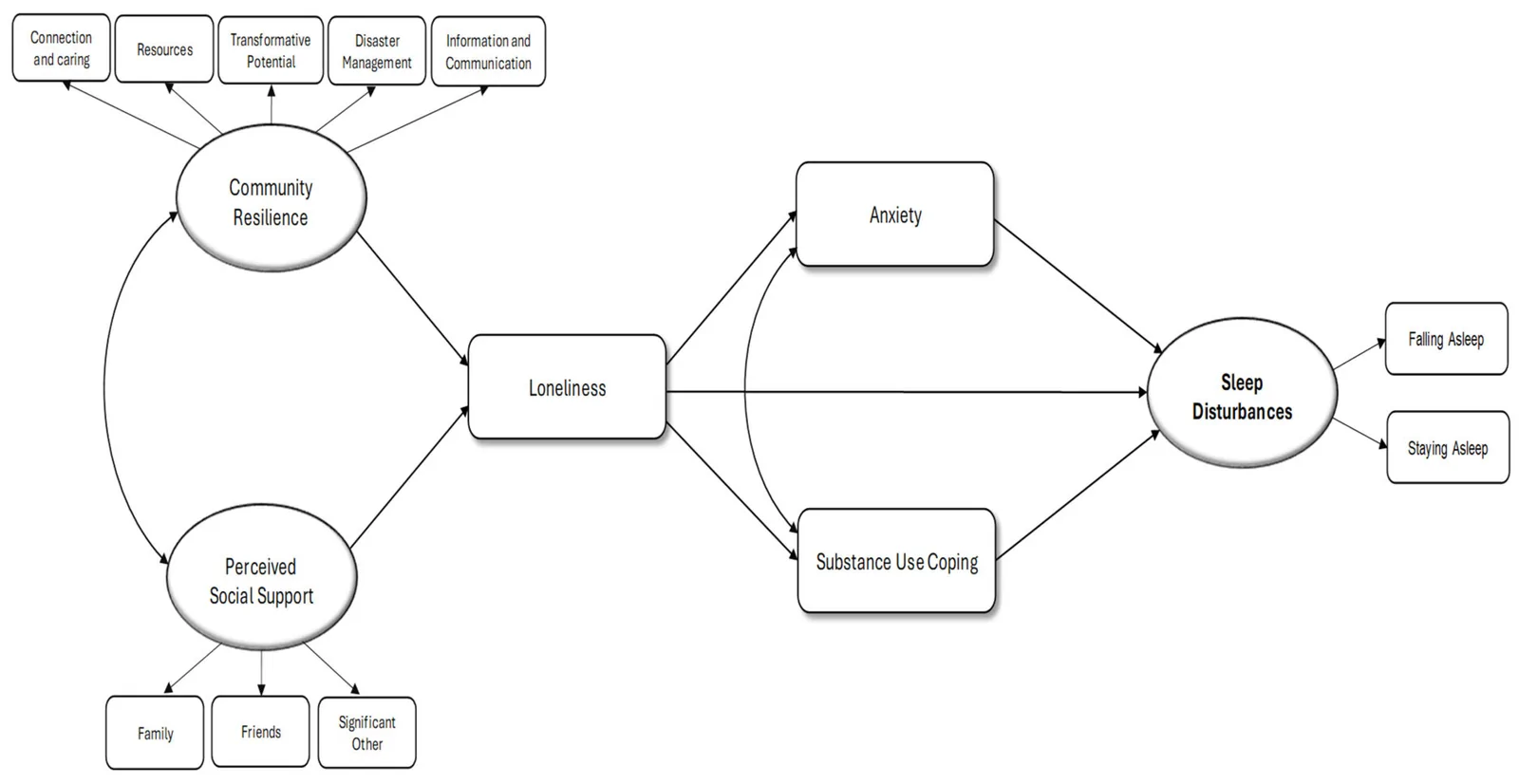
Proposed structural equation model of anxiety and substance use coping as potential mediators between loneliness and sleep disturbances.

The purpose of this study was to examine the relationship between loneliness and sleep disturbances and explore the roles of community resilience and psychosocial factors (social support, anxiety, and substance use coping) in shaping this relationship. Guided by previous studies [7,16,18,21,22,27,28], the current study developed and tested two hypothesized models. In Model 1, loneliness is viewed as a direct predictor of sleep disturbances without the inclusion of mediating pathways (Figure 1). It was hypothesized that loneliness would be directly and positively associated with sleep disturbances. Community resilience and perceived social support, as broad social-contextual factors recognized to protect against loneliness [22,27], were hypothesized to be directly and inversely related to loneliness. In Model 2, anxiety and substance use coping are included as potential mediators of the relationship between loneliness and sleep disturbances, while still accounting for the direct pathway (Figure 2). It was hypothesized that loneliness would be directly and positively associated with sleep disturbances; in addition, these relationships would be indirect through potential mediating variables of anxiety and substance use coping. As in Model 1, community resilience and perceived social support were hypothesized to be directly and inversely related to loneliness. There would be correlations between community resilience and perceived social support, and between anxiety and substance use coping.

Community resilience is represented by five dimensions: connection and caring, resources, transformative potential, disaster management, and information and communication [29]. Perceived social support is represented by three dimensions: family, friends, and significant other [30,31]. Sleep disturbances are represented by two components: falling asleep and staying asleep.

## 2. Methods

### 2.1. Sampling Procedures

The data used in this analysis were collected in the fall of 2018. Using public domain parcel maps, the current study utilized ArcGIS 10.2 to randomly select households located between Interstate 10 and the Gulf of Mexico coastline across three Mississippi counties: Hancock, Harrison, and Jackson. The inclusion criteria of the study required participants to be residents living within an officially sampled household situated between Interstate 10 and the Gulf of Mexico coastline across Hancock, Harrison, or Jackson County, and at least 18 years of age at the time of administration. Exclusion criteria included individuals who (1) were unable to provide informed consent due to cognitive impairment or a severe language barrier (as the survey was administered in English), (2) resided in non-residential structures or oversized parcels (>2 acres) excluded during the initial geographic GIS filtering, or (3) were temporary visitors or tourists who did not permanently reside within the three coastal Mississippi counties. Surveyors, primarily graduate and undergraduate students from a public university in Mississippi, worked in teams of two or more and were assigned specific geographic areas to survey. Each team was then given an aerial map laid out in a grid pattern with selected households highlighted. All surveyors were required to attend training to ensure proper survey administration and competence using the survey maps. The project was reviewed and approved by the Institutional Review Board at the University affiliated with the researchers. Participation in the survey was entirely voluntary, and individuals could choose to withdraw or decline to answer any question at any point without penalty. Completed questionnaires were assigned unique alphanumeric identification codes for data entry and analysis. All digital datasets were stored on password-protected, encrypted secure servers accessible only to the research team.

### 2.2. Measures

#### 2.2.1. Community Resilience

Community resilience was assessed by the self-reported measure of the Communities Advancing Resilience Toolkit (CART). The CART consists of 24 items reflecting dimensions of community resilience such as connection and caring, resources, transformative potential, disaster management, and information and communication [29]. Through a series of exploratory factor analysis and confirmatory factor analyses, Pfefferbaum et al. [29] identified a five-factor model of community resilience including (1) connection and caring (5 items, e.g., “People in my community help each other”), (2) resources (5 items, e.g., “My community has the resources it needs to take care of community problems (resources include money, information, technology, tools, raw materials, and services)”), (3) transformative potential (6 items, e.g., “My community looks at is successes and failures so it can learn from the past”), (4) disaster management (4 items, e.g., “My community can provide emergency services during a disaster”), and (5) information and communication (4 items, e.g., “My community keeps people informed (via TV, radio, newspaper, Internet, phone, and neighbors) about issues that are relevant to them”).

Participants were asked to rate on a 5-point Likert scale, ranging from one (strongly disagree) to five (strongly agree). Evidence of adequate reliability for each dimension of the CART was reported [29]. In this study, Cronbach’s alphas were 0.89 for connection and caring, 0.84 for resources, 0.92 for transformative potential, 0.87 for disaster management, and 0.86 for information and communication.

#### 2.2.2. Perceived Social Support

The Multidimensional Scale of Perceived Social Support (MSPSS) was used to measure perceived social support. It is a 12-item self-reported measure designed to assess an individual’s perceptions of three sources of social support, including family, friends, and significant others [31]. Responses were coded on a 7-point Likert scale, ranging from one (very strongly disagree) to seven (very strongly agree), with higher scores indicating higher levels of perceived social support. Evidence of reliability and validity for the MSPSS has been reported in previous studies [31,32]. In this study, Cronbach’s alphas were 0.90 for family, 0.95 for friends, and 0.90 for significant others.

#### 2.2.3. Loneliness

The UCLA 3-item Loneliness scale was used to measure subjective feelings of loneliness and social isolation [33]. The scale asks how often respondents “feel they lack companionship,” feel left out,” and “feel isolated from others” [33]. The scale has shown adequate internal consistency and validity [33]. Participants rated each statement on a three-point scale ranging from 1 (hardly ever) to 3 (often). A sum score was computed from the three items, with higher scores reflecting greater loneliness. The Cronbach’s alpha in the current study was 0.82.

#### 2.2.4. Anxiety

The 7-item Generalized Anxiety Scale (GAD-7) was designed to measure the severity of anxiety [34]. Kroenke et al. [35] identified the first two items of the GAD-7 as core components of any anxiety disorder (GAD-2): (1) “Feeling nervous, anxious, or on edge” and (2) “Not being able to stop or control worrying.” The psychometric properties of GAD-2 have been tested and validated in several studies [35,36,37]. The current study used the GAD-2. Participants rated each item on a scale from 0 (not at all) to 3 (nearly every day), and the scores were summed up to create a total anxiety score, with higher scores indicating greater anxiety. GAD-2 has been proven to be a reliable and valid measure for anxiety in previous studies [35,38]. For the two congeneric items, the Spearman–Brown coefficient was used for testing reliability, and the score was 0.80.

#### 2.2.5. Substance Use Coping

The 28-item Brief COPE Inventory was designed to assess how people cope with stressful life events. This inventory includes 14 subscales (e.g., active coping, positive reframing, substance use) composed of 2 items each [39]. The psychometric properties of the Brief COPE Inventory have been tested and validated in previous studies [39,40]. In the current study, the substance use coping subscale was used: (1) “I’ve been using alcohol or other drugs to make myself feel better” and (2) “I’ve been using alcohol or other drugs to help me get through stressful situations.” Participants rated the items on a 4-point Likert scale ranging from 0 (not at all) to 3 (a lot). The scores were summed to create a total score, with higher scores indicating greater reliance on substance use for coping. For the two congeneric items, the Spearman–Brown coefficient was used for testing reliability, and the score was 0.88.

#### 2.2.6. Sleep Disturbances

Two items were created to measure sleep disturbances: (1) “I have problems going to sleep” and (2) “I have problems staying asleep.” Respondents were asked to rate on a 5-point Likert scale, ranging from 1 (“Strongly Disagree”) to 5 (“Strongly Agree”), with higher scores indicating greater sleep disturbances. For the two congeneric items, the Spearman–Brown coefficient was used for testing reliability, and the score was 0.82.

### 2.3. Statistical Analysis

The proposed structural equation model (SEM) was tested using AMOS (version 30) [41]. A two-step modeling procedure was applied to test the proposed model. A confirmatory factor analysis (CFA) was conducted first to ensure whether observed factors reflect latent constructs suitably [42], followed by testing the full SEM. Full information maximum likelihood in AMOS was applied to treat missing data. Several indices were used to assess model fit: the chi-square test (χ^2^), the normed fit index (NFI), the Tucker–Lewis index (TLI), the comparative fit index (CFI), and the root mean squared error of approximation (RMSEA) with a 90% confidence interval (CI). Nonsignificant chi-square, the NFI, TLI, and CFI of no less than 0.95, and the RMSEA of no more than 0.06 typically suggest a good fit [43]. Because the chi-square statistic is sensitive to sample size, the normed chi-square ratio, χ^2^/df, was used as a supplementary indicator of model fit. Values less than 3 were interpreted as indicating acceptable model fit [44]. In addition, the Sobel test was used to determine whether the indirect effect of loneliness on sleep disturbances through the mediating variables of anxiety and substance use coping [45].

## 3. Results

### 3.1. Sample Characteristics

A total of 310 participated in the survey. Approximately 53% of the participants were female, and 65% were White/Non-Hispanic. The average age of the participants was 50.47 years (SD = 17.57). Among the respondents, almost 30% of them had completed high school or less, 56% had some college/associate’s/bachelor’s degree, while only 15% had a master’s degree or higher. In terms of household income, approximately 19% of respondents reported earning less than $20,000, 23% reported incomes between $20,000 and $39,999, 20% reported incomes between $40,000 and $59,999, and 38% reported earning $60,000 or more. Table 1 presents descriptive statistics of sample characteristics.

### 3.2. Bivariate Analyses

Bivariate analyses using Spearman’s rank correlation coefficient were conducted to examine associations among the variables, with results presented in Table 2. Demographic factors, including gender, age, ethnicity (White/Non-Hispanic vs others), education, and income, were assessed as potential confounders. Because none of these variables showed a strong correlation with the endogenous outcomes, they were not included as controls in the SEM. Sleep disturbances were significantly associated with loneliness and anxiety, whereas their associations with substance-use coping were very weak.

### 3.3. Measurement Model Test

Before testing the full structural equation model, a separate confirmatory factor analysis (CFA) was performed to confirm the validity of the measurement model. Measure fit statistics and factor loadings were examined: *χ*^2^ (32, N = 310) = 77.11, *p* < 0.001, χ^2^/df = 2.41, NFI = 0.95, TLI = 0.95, CFI = 0.97, RMSEA = 0.07 (90% CI [0.05, 0.09]), a probability of close fit (PCLOSE) = 0.07, suggesting that the overall model fit ranges from reasonable to marginally acceptable. All observed factors loaded significantly on their respective constructs (*p* < 0.01). The factor loadings of Connection and Caring, Resources, Transformative Potential, Disaster Management, and Information and Communication on Community Resilience were 0.71, 0.81, 0.89, 0.78, and 0.80, respectively. The factor loadings of Family, Friends, and Significant Others to Perceived Social Support were 0.84, 0.73, and 0.79, respectively. The factor loadings of Falling Asleep and Staying Asleep to Sleep Disturbances were 0.93 and 0.74, respectively.

### 3.4. Full Model Test

**Figure 3:**
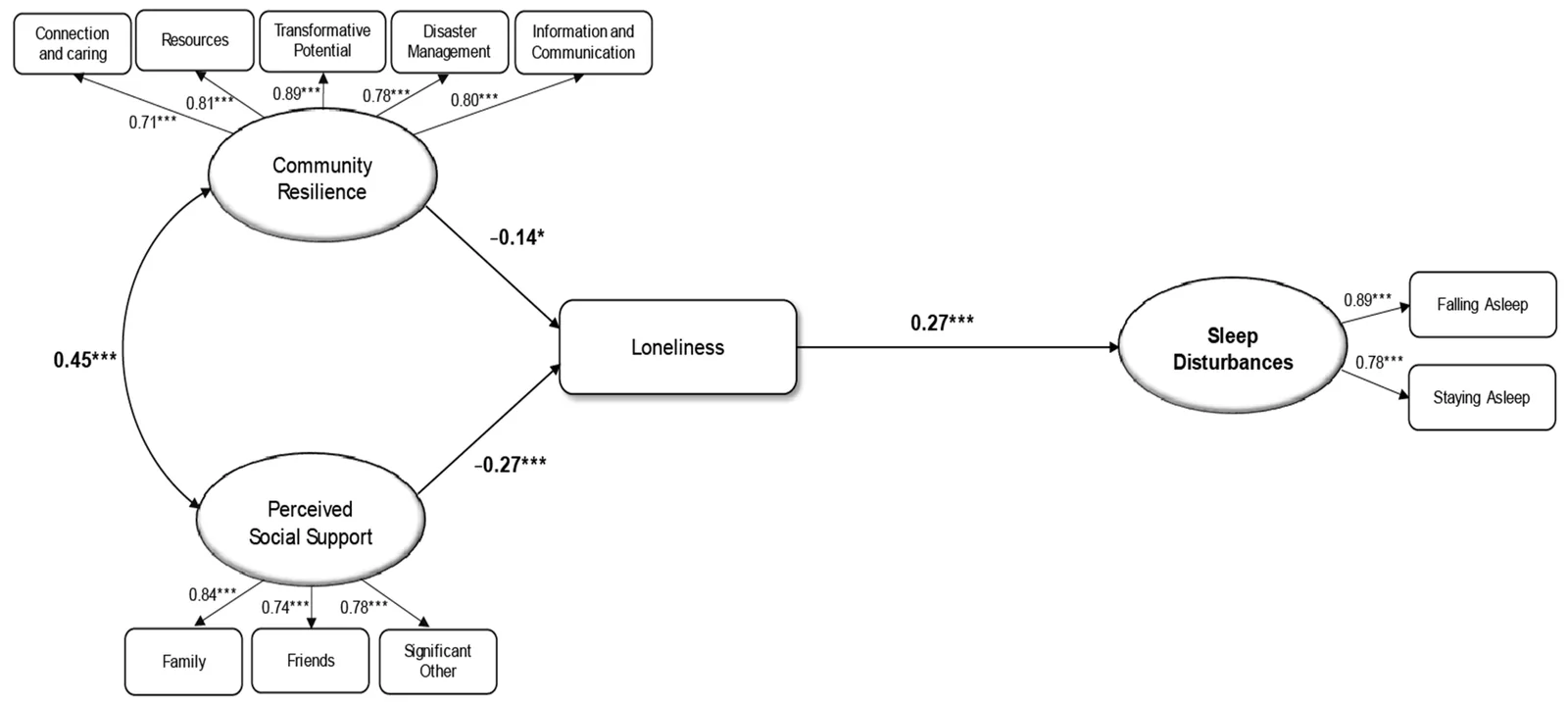
Structural equation model of loneliness and sleep disturbances. Note: *χ*^2^ (41, N = 310) = 84.49, *p* < 0.001, χ^2^/df = 2.06, NFI = 0.95, TLI = 0.95, CFI = 0.97, RMSEA = 0.06 (90% CI [0.04, 0.08]), a probability of close fit (PCLOSE) = 0.20. Standardized coefficients are presented; * *p* < 0.05; *** *p* < 0.001.

First, this study tested the proposed structural equation model without the potential mediating variables of anxiety and substance use coping using maximum-likelihood estimation in AMOS (Version 30). This hypothesized model yielded the following fit indices: *χ*^2^(41, N = 310) = 84.49, *p* < 0.001, χ^2^/df = 2.06, NFI = 0.95, TLI = 0.95, CFI = 0.97, RMSEA = 0.06 (90% CI [0.04, 0.08]), a probability of close fit (PCLOSE) = 0.20. Overall, these indices suggested a reasonable to marginally acceptable fit to the sample data. The path diagram of the model with standardized parameter estimates appears in Figure 3. As expected, loneliness was significantly associated with sleep disturbances, such that higher levels of loneliness were linked to greater sleep disturbance (*β* = 0.27, *p* < 0.001).

**Figure 4:**
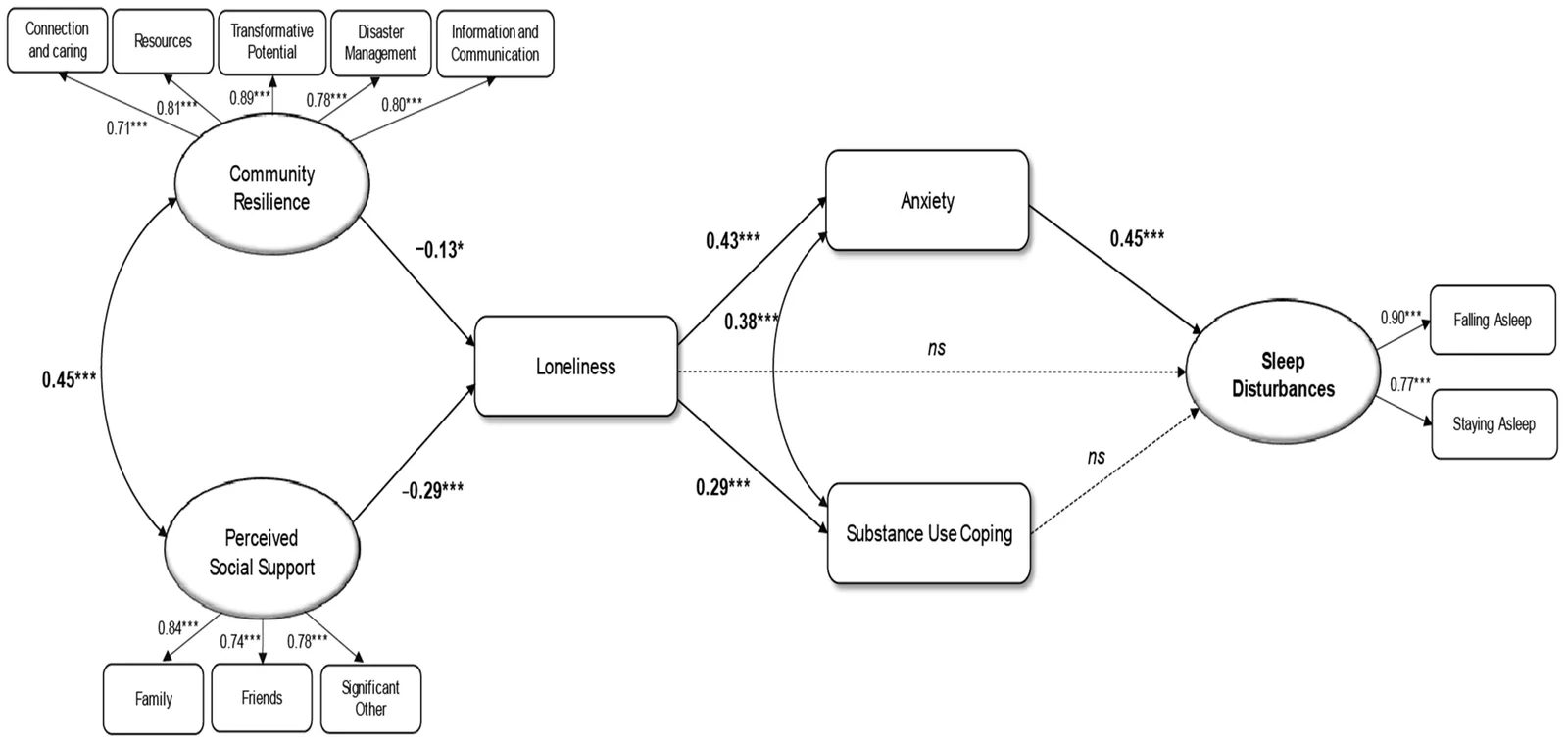
Structural equation model of anxiety and substance use coping as potential mediators between loneliness and sleep disturbances. Note: *χ*^2^ (59, N = 310) = 103.38, *p* < 0.001, χ^2^/df = 1.75, NFI = 0.94, TLI = 0.96, CFI = 0.97, RMSEA = 0.05 (90% CI [0.03, 0.07]), a probability of close fit (PCLOSE) = 0.51. Standardized coefficients are presented; *ns* denotes non-significant paths, represented by dotted lines; * *p* < 0.05; *** *p* < 0.001.

Subsequently, the potential mediators of anxiety and substance use coping were integrated into the model to evaluate their effects This hypothesized model yielded the following fit indices: *χ*^2^ (59, N = 310) = 103.38, *p* < 0.001, χ^2^/df = 1.75, NFI = 0.94, TLI = 0.96, CFI = 0.97, RMSEA = 0.05 (90% CI [0.03, 0.07]), a probability of close fit (PCLOSE) = 0.51. Overall, these indices suggested a reasonable to marginally acceptable fit to the sample data. However, the NFI (0.94) fell slightly below the conventional cutoff of 0.95, indicating that the model’s fit should not be considered optimal. The path diagram of the model with standardized parameter estimates appears in Figure 4. Both community resilience and perceived social support were significantly associated with loneliness, such that higher levels of each were linked to lower levels of loneliness (*β* = −0.14, *p* = 0.037; *β* = −0.27, *p* < 0.001, respectively). Once the potential mediators were included, the direct effect of loneliness on sleep disturbances became non-significant; instead, it was indirect through anxiety. In other words, higher levels of loneliness were associated with increased anxiety symptoms (*β* = 0.43, *p* < 0.001), which, in turn, were linked to greater sleep disturbances (*β* = 0.45, *p* < 0.001). This indirect effect was statistically significant (Sobel z test = 4.77, *p* < 0.001). On the other hand, while higher levels of loneliness were associated with increased substance use coping (*β* = 0.29, *p* < 0.001), no significant relationship was found between substance use coping and sleep disturbances (*β* = 0.01, *p* = 0.872). In addition, the relationships between community resilience, social support, and loneliness were examined. Both community resilience and perceived social support were significantly associated with loneliness, such that higher levels of each were linked to lower levels of loneliness (*β* = −0.13, *p* = 0.046; *β* = −0.29, *p* < 0.001, respectively). As expected, there were significant associations between community resilience and perceived social support (*r* = 0.45, *p* < 0.001) and between anxiety and substance use coping (*r* = 0.38, *p* < 0.001). The squared multiple correlation coefficient (*R*^2^) for sleep disturbances in the full model was 0.24, indicating that the model variables accounted for 24% of the variance in sleep disturbances.

## 4. Discussion

With a sample of 310 residents in three coastal counties in Mississippi, the current study tested hypothesized models in which loneliness has a direct relationship with sleep disturbances and an indirect relationship through the potential mediating variables of anxiety and substance use coping. Initially, a significant direct relationship was observed between loneliness and sleep disturbances. However, this direct path became non-significant upon the inclusion of anxiety and substance use coping, shifting instead to an indirect pathway through anxiety. Interestingly, although lonelier individuals were more likely to engage in substance use coping, the coping strategy was not significantly related to sleep disturbances within the mediation model, which is inconsistent with prior research supporting the influence of substance use on sleep architecture [18,19]. One possible explanation is that the substance use coping measure captured the tendency to rely on substances as a coping strategy, but did not assess the frequency, quantity, timing, or type of alcohol or drug use, factors that may have differential effects on sleep architecture [19,46]. Future research should incorporate more detailed measures of substance use, including its frequency, quantity, timing, and type, to better capture its potential impact on sleep. The indirect pathway from loneliness to sleep disturbances through anxiety is consistent with prior research [13,47]. Additionally, findings of the current study highlight the roles of community resilience and social support in mitigating individual loneliness, which is consistent with prior research [27].

The significant Sobel test and the non-significant direct path following its inclusion suggest that anxiety may serve as a mediating role linking loneliness and sleep disturbance, which offers an understanding of the internalizing process of social isolation. While loneliness is cross-sectionally associated with sleep vulnerability, the results suggest it is the subsequent psychological distress, especially the cognitive and physiological arousal associated with anxiety, that is closely related to sleep process disruption. This aligns with the evolutionary theory of loneliness, which posits that perceived social isolation may correlate with a state of hyper-vigilance for self-preservation. In this state, the brain is hypothesized to remain in a high-alert phase, manifesting as the anxiety symptoms observed in our model, which statistically patterns with a difficult transition into deep, restorative sleep [48]. However, given the cross-sectional nature of this study, these pathways reflect structural associations rather than directional processes, and a bidirectional relationship where sleep disturbances exacerbate anxiety cannot be ruled out. This shift from a direct path to an indirect pathway tentatively suggests, for future longitudinal tracking, that clinical interventions focusing solely on increasing social outings may be insufficient if the concurrent anxiety remains unaddressed.

Furthermore, while both community resilience and perceived social support are inversely related to loneliness, the direct path from community resilience is notably modest (*β* = −0.13, *p* = 0.046). This indicates that community-level infrastructure explains only a small portion of individual isolation, and we must be careful not to overstate it as a primary clinical cure. However, from a public health framework, small effect sizes targeting upstream environmental factors can still show meaningful population-level benefits. While individual therapies have larger effects, they are resource-intensive and reach few people, while a modest protective effect from community infrastructure, such as robust local communication networks and neighborhood support, operates continuously across an entire population. Therefore, while community resilience should be viewed as a supplementary structural target rather than a singular solution, its scalability makes it a highly efficient public health investment alongside individual social support and clinical anxiety treatments. While social support has long been recognized as a factor, the inclusion of community resilience provides a broader lens for public health. Strengthening local networks and disaster management protocols may yield dividends in the form of improved psychological well-being and better sleep health for residents.

The structural pathways identified in this pre-pandemic baseline carry profound implications for today’s post-pandemic landscape. While the overall prevalence of psychological distress has risen, the underlying structural architecture remains unchanged. For instance, our baseline path from loneliness to anxiety (*β* = 0.43) is likely to be even more pronounced now, as remote work and fractured neighborhood networks have institutionalized social isolation [49]. Furthermore, because the pandemic severely tested local communication and governance channels, community resilience has transitioned from a theoretical buffer to an absolute logistical necessity. These findings show that post-pandemic public health strategies cannot focus on sleep in isolation; instead, they must invest in community-level strengths to reduce loneliness and protect population health. These findings highlight the need for multilevel strategies to improve sleep quality, which creates a nested system in which community strengths and individual support systems jointly protect against isolation, alongside clinical approaches to alleviate anxiety.

### Limitations and Future Research

While these findings are significant, several limitations must be mentioned. First, this study utilized a cross-sectional sampling approach. As a result, the temporal ordering among loneliness, anxiety, and sleep disturbances cannot be established, leaving the structural model vulnerable to reverse causality. Future longitudinal research might clarify the causal processes leading to sleep disturbances. Second, the sampling framework was limited to households located between Interstate 10 and the Gulf coastline in three Mississippi counties, which may limit the generalizability of the findings, even within Mississippi. Future studies should replicate this research using more representative random samples across Mississippi and the United States.

Third, the measurement of key constructs relied on extremely short forms, including the GAD-2 for anxiety and a 2-item self-report scale for sleep disturbances. While these brief instruments are highly efficient and necessary for minimizing participant burden in large-scale studies, they cannot capture the full clinical depth or diagnostic nuances of sleep pathology. Consequently, the pathways in our structural model represent broad psychosocial associations rather than definitive clinical diagnoses, and the model’s parameters should be treated as population-level indicators rather than diagnostic tools [50].

Fourth, community resilience was measured based on individual perceptions, which may not fully capture the objective availability or quality of community characteristics. Future research would benefit from incorporating physical infrastructure into assessments of community resilience [51].

Fifth, to further explore the roles of community resilience and perceived social support in loneliness and sleep, future research could examine whether community resilience and perceived social support moderate the association between loneliness and sleep disturbances, such that this association varies depending on the levels of these protective factors.

Sixth, overall, the hypothesized models demonstrated a reasonable to marginally acceptable fit across most indices. However, the NFI for the model including anxiety and substance use coping as potential mediators was 0.94, slightly below the conventional cutoff of 0.95, indicating that this model’s fit should not be considered optimal. In addition, although the final model explained 24% of the variance in sleep disturbances, 76% of the variance remained unexplained, suggesting that additional unmeasured confounders or alternative pathways may be involved. Future research should examine how physical health conditions and additional mental health conditions influence sleep, as well as how lifestyle factors (e.g., physical activity) and life stressors (e.g., financial strain and employment changes) shape these associations. These factors have been identified in previous studies as important contributors to understanding sleep [52,53]. Therefore, the findings from the current model should be interpreted with caution, and future research should replicate this model using larger, more diverse samples while incorporating these additional variables to further evaluate its robustness and provide a more comprehensive understanding of sleep disturbances. Seventh, because all constructs were measured concurrently using self-reported questionnaires, the data are susceptible to common method bias, which can artificially inflate the strength of observed relationships. Future research would benefit from utilizing multi-source or multi-method designs to completely decouple measurement methods.

## 5. Conclusions

This study examined the relationship between loneliness and sleep disturbances among residents of three coastal Mississippi counties, and the roles that community resilience and psychosocial factors play in shaping this relationship. The findings showed that loneliness does not happen in isolation: it is also shaped by a wider network of protective and risk factors. While an initial direct relationship between loneliness and sleep disturbances was observed, this relationship was no longer significant after anxiety and substance-use coping were added into the model; only anxiety remained a significant predictor, which means that the psychological distress with loneliness may be the more potent driver of sleep impairment. In contrast, substance use coping, although associated with loneliness, did not carry forward to sleep disturbances, which suggests that this coping pathway operates somewhat independently of the sleep process examined here.

The study also reinforced the protective roles of community resilience and perceived social support, both of which were inversely related to loneliness. These findings suggest that efforts to improve sleep health in vulnerable populations cannot rely only on individual-level clinical interventions, such as anxiety treatment or sleep hygiene programs, but must also be paired with community-level investments that strengthen local networks, communication infrastructure, and disaster preparedness. By situating individual psychological processes within their broader community context, this study contributes to a more integrated, multilevel understanding of sleep health, one in which clinical and community-based strategies are not competing approaches but complementary components of a shared protective system.

Future research building on a longitudinal design would help clarify the directionality of these pathways and further establish whether strengthening community resilience produces measurable improvements in sleep health over time.

## Figures and Tables

**Table 1:** Descriptive statistics of the study sample (n = 310).

Variable	N (%)	Mean (SD)
Gender		
Male	141 (46.69%)	
Female	161 (53.31%)	
Age		50.47 (17.57)
White/Non-Hispanic		
Yes	197 (65.23%)	
No	105 (34.77%)	
Education		
High school or less	90 (29.32%)	
Some college/Associate’s/Bachelor’s degree	172 (56.03%)	
Master’s or higher	45 (14.66%)	
Income		
Less than or equal to $20,000	52 (18.9%)	
$20,000 to $39,999	64 (23.3%)	
$40,000 to $59,999	54 (19.6%)	
$60,000 to $79,999	40 (14.5%)	
$80,000 to $99,999	27 (9.8%)	
Community Resilience		
Connection and Caring		19.54 (4.02)
Resources		18.09 (3.96)
Transformative Potential		21.87 (4.71)
Disaster Management		15.23 (3.19)
Information and Communication		15.36 (3.35)
Perceived Social Support		
Family		23.03 (5.41)
Friends		22.71 (5.76)
Significant Other		23.42 (5.49)
Loneliness		4.14 (1.64)
Substance Use Coping		2.85 (1.60)
Anxiety		0.90 (1.53)
Sleep Disturbances		
Falling Asleep		2.30 (1.43)
Staying Asleep		2.42 (1.42)

**Table 2:** Bivariate statistics of study variables.

	1	2	3	4	5	6	7	8	9	10	11	12	13	14	15	16	17	18
1. Gender	─																	
2. Age	0.02	─																
3. White/ Non-Hispanic	0.02	−0.03	─															
4. Education	0.03	0.06	−0.30 ***	─														
5. Income	0.01	0.15 *	−0.23 ***	0.41 ***	─													
6. CR: Connection and Caring	0.09	0.21 ***	−0.07	0.04	0.17 **	─												
7. CR: Resources	0.06	0.09	−0.15 *	0.05	0.19 **	0.61 ***	─											
8. CR: Transformative Potential	0.06	0.10	−0.14 *	0.09	0.16 **	0.59 ***	0.75 ***	─										
9.CR: Disaster Management	0.10	0.06	−0.24 ***	0.15 *	0.17 **	0.53 ***	0.57 ***	0.65 ***	─									
10. CR: Information and Communication	0.09	0.09	−0.06	0.11	0.18 **	0.55 ***	0.53 ***	0.66 ***	0.67 ***	─								
11. PSS: Family	0.13 *	0.09	−0.12 *	0.17 **	0.37 ***	0.32 ***	0.41 ***	0.36 ***	0.30 ***	0.34 ***	─							
12. PSS: Friends	0.15 *	0.12 *	−0.16 **	0.10	0.30 ***	0.34 ***	0.31 ***	0.35 ***	0.36 ***	0.39 ***	0.68 ***	─						
13. PSS: Significant Other	0.16 **	0.15 *	−0.20 ***	0.17 **	0.31 ***	0.32 ***	0.31 ***	0.32 ***	0.26 ***	0.34 ***	0.73 ***	0.64 ***	─					
14. Loneliness	−0.09	−0.02	0.07	−0.16 **	−0.41 ***	−0.25 ***	−0.22 ***	−0.27 ***	−0.17 **	−0.20 ***	−0.34 ***	−0.34 ***	−0.28 ***	─				
15. Anxiety	0.01	−0.14 *	0.02	−0.09	−0.20 ***	−0.18 **	−0.12 *	−0.08	−0.08	−0.10	−0.15 *	−0.15 *	−0.05	0.45 ***	─			
16. Substance Use Coping	−0.13 *	−0.07	−0.02	−0.06	−0.04	−0.02	−0.01	−0.04	−0.05	−0.03	−0.09	−0.05	−0.06	0.24 ***	0.34 ***	─		
17. SD: Falling Asleep	0.01	−0.05	−0.09	0.05	−0.10	−0.21 ***	−0.13 *	−0.14 *	−0.10	−0.15 **	−0.10	−0.18 **	−0.10	0.26 ***	0.39 ***	0.18 **	─	
18. SD: Staying Asleep	0.05	0.02	−0.19 **	0.04	−0.03	−0.18 **	−0.12 *	−0.10	−0.07	−0.13 *	−0.07	−0.13 *	−0.06	0.23 ***	0.38 ***	0.10	0.72 ***	─

Note: CR: Community Resilience; PSS: Perceived Social Support; SD: Sleep Disturbances; * *p* < 0.05; ** *p* < 0.01; *** *p* < 0.001.

## Data Availability

The data presented in this study are available on request from the corresponding author. The data are not publicly available because the original study protocol and participant consent process did not include provisions for depositing the data in a public repository. Although the dataset has been anonymized, public sharing would therefore go beyond the scope of the consent and data-management arrangements under which the data were collected.
